# Effect of Early-Life Treatment of Piglets with Long-Acting Ceftiofur on Colonization of *Streptococcus suis* Serotype 7 and Elicitation of Specific Humoral Immunity in a Farm Dealing with Streptococcal Diseases

**DOI:** 10.3390/pathogens7020034

**Published:** 2018-03-29

**Authors:** Christine Unterweger, Ursula Ruczizka, Joachim Spergser, Christoph Georg Baums, Isabel Hennig-Pauka

**Affiliations:** 1 University Clinics for Swine, University of Veterinary Medicine Vienna, Veterinaerplatz 1, 1210 Vienna, Austria; ursula.ruczizka@vetmeduni.ac.at (U.R.); isabel.hennig-pauka@vetmeduni.ac.at (I.H.-P.); 2 Institute of Microbiology, University of Veterinary Medicine Vienna, Veterinaerplatz 1, 1210 Vienna, Austria; joachim.spergser@vetmeduni.ac.at; 3 Institute for Bacteriology and Mycology, Centre for Infectious Diseases, Faculty of Veterinary Medicine, Leipzig, An den Tierkliniken 19, 04103 Leipzig, Germany; Christoph.baums@vetmed.uni-leipzig.de

**Keywords:** early life ceftiofur treatment, *streptococcus suis serotype* 7, transmission, colonization, pig, bactericidal killing assay, antibodies, drug resistance

## Abstract

In newborn piglets treatment with long-acting ceftiofur is a common approach to reduce losses due to streptococcal diseases on farms, even if problems start after weaning. The purpose of this study was to examine the influence of a single early-life treatment on *Streptococcus (S.) suis* colonization, transmission, immunoreaction, and drug resistance over an observation period of 14 weeks. In a farm with a history of streptococcal disease and isolation of a *S. suis cps* 7 *mrp*+, *arc*A+ isolate from diseased piglets, half of each litter was treated with a long-acting ceftiofur on day 1. *S. suis*-isolates were profiled and serum samples were tested for opsonizing antibodies. Treated and untreated pigs did not differ according to average daily weight gains, *S. suis*-isolation rates and level of opsonizing antibodies. Although the invasive *cps* 7 strain was not detected in a single piglet over 14 weeks, all animals developed bactericidal activity. No resistance to ceftiofur, but resistance to tetracyclins (100%), and trimethoprim/sulfamethoxazole (53%) was shown. Our results indicate that early treatment with ceftiofur does not prevent colonization and transmission of *S. suis* or the induction of bactericidal humoral immunity in nursery and fattening pigs. The necessity of continuous usage should be reconsidered.

## 1. Introduction

*Streptococcus (S.) suis* was isolated for the first time in the early 1950s in Europe during a disease outbreak in swine [1,2]. Nowadays, this pathogen can be considered as the most important bacterial cause for meningitis in pigs worldwide. *S. suis* is characterized by a high diversity of strains and 35 different serotypes are known so far, classified according to their capsular polysaccharides [3]. Next to meningitis, a wide variety of additional pathologies in piglets of different age groups, such as arthritis, (poly)serositis, endocarditis, otitis media, and bronchopneumonia lead to high economic losses in pig production [4]. *S. suis* is a very successful colonizer of mucosal surfaces and is considered as part of the physiological pig’s upper respiratory flora [5]. With isolation rates as high as 98% [6], clinically healthy piglets and sows carry multiple serotypes of *S. suis* in their tonsillar crypts and, therefore, play a key role in the epidemiology of *S. suis*-associated diseases [7,8,9]. Piglets can become colonized during parturition when they get in contact and/or swallow *S. suis* from sow vaginal secretions during passage of the birth channel [10,11,12]. Colonized piglets carrying pathogenic *S. suis* strains in their upper respiratory tract might be able to infect the whole group by nose to nose contact or by aerosol transmission [13,14]. Streptococcal diseases usually peak in the nursery some weeks after weaning, after intense mixing of piglets, when maternal immunity has declined after the first 3–5 weeks of life [15,16] and the adaptive immune response of the piglets was not complete [17]. However, in general, pigs of any age can be affected [17,18]. Severity of disease or morbidity depends on the serotype and virulence factors as differences in virulence among strains are well recorded [19,20,21,22]. In Europe, *cps* 1, 2, 7, and 9 are more common among invasive isolates than any of the other 31 serotypes [23,24]. *Cps* 7 infections have been associated with severe herd problems of meningitis and arthritis in the nursery and in growers [22,25,26,27]. Control of *S. suis* infections is a difficult challenge for farmers and swine practitioners because of the high colonization rate and often unsuccessful vaccination attempts [28]. Instead of vaccination, metaphylactic treatment of newborn piglets with antimicrobials is routinely used in order to reduce colonization and transmission in piglets, as well as the bacterial burden in a herd [29]. Ceftiofur is a third-generation cephalosporin developed strictly for veterinary use and licensed for the treatment of bacterial respiratory diseases in swine and cattle [30]. Due to its pharmacodynamic and pharmacokinetic properties and a broad spectrum of activity against Gram-positive and Gram-negative bacteria [31], ceftiofur is used quite often in case of streptococcal infections, though 3rd and 4th generation cephalosporins are on the WHO- list of critically-important antimicrobials [32]. The aim of this study was to assess the effect of a single early-life ceftiofur treatment on *S. suis* colonization of the tonsils, selection of antimicrobial resistance, induction of opsonizing antibodies as determined in bactericidal assays and blood cell counts, as well as on *S. suis* transmission between pen-mates in the nursery and early fattening over a 14 week period in a farm dealing with *S. suis cps* 7 associated problems. Our results indicate that early treatment with ceftiofur in piglets does not necessarily prevent colonization and transmission of *S. suis.* This is in contrast to the results of Gascho et al. [33], who described a significant reduction in carrier sows and weaned carrier piglets when treating pre-farrowing sows with the same long acting ceftiofur preparation. We also showed an induction of bactericidal humoral immunity against the virulent *cps* 7 strain in nursery and fattening pigs of both groups despite of missing clinical signs during this trial, highlighting the importance of biosecurity and management measures in case of *S. suis* associated disease outbreaks. No changes in antimicrobial resistance patterns could be seen over the study period. Anyway, veterinarians and farmers should critically re-evaluate their continuous use of antimicrobials.

## 2. Results

### 2.1. Streptococcal Diseases on Farm Prior to the Beginning of the Study

**Figure 1:**
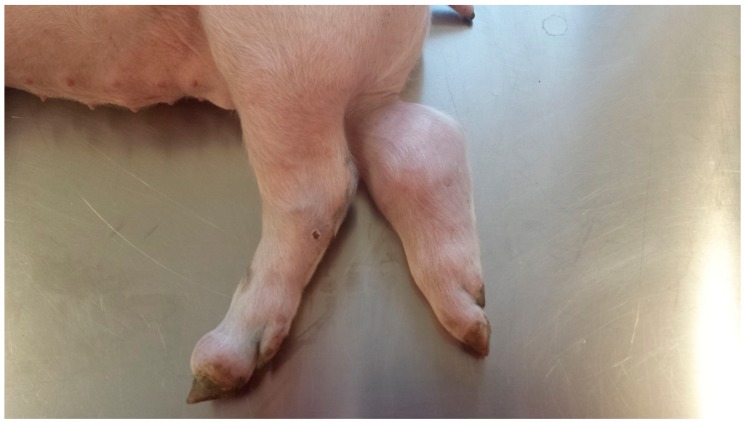
High-graded purulent polyarthritis in 6–7 week-old piglet caused by *Streptococcus suis cps* 7 *(mrp+*, *arc*A+, *sly−)*.

The study was performed in a farrow to finish farm—unsuspicious for Porcine Reproductive and Respiratory Disease Virus—with 140 sows (Large White) with a history of a *S. suis cps* 7 herd problem over months. Periodically, streptococcal disease started in the 3rd week of life and peaked in seven to nine weeks old piglets in the nursery with 15% morbidity and approximately 4–6% mortality despite antimicrobial and antiphlogistic treatment. Diseased piglets showed various clinical signs, like swollen joints (Figure 1) often associated with lameness and central nervous disorders with paddling in lateral recumbency and nystagmus. 

Gross pathological findings during necropsy of diseased piglets were purulent leptomeningitis and purulent arthritis. Synovia and cerebrospinal fluid or brain tissue samples (*n* = 7) collected from five untreated piglets suffering from severe disease were positive for *S.suis* and further characterized by multiplex PCR, conducted as previously described [24], showing profiles of virulence associated genes as specified in Table 1 (capsular polysaccharides *cps*, muramidase-released protein *mrp*, arginine deiminase *arc*A, suilysin *sly* and extracellular factor *epf*)*.* All seven isolates were resistant to tetracycline, but sensitive to amoxicillin, cefquinome, ceftiofur, lincospectin, tulathromycin, tilmicosin, and sulfamethoxazole-trimethoprim.

### 2.2. Clinical Findings, Body Weight Gain, Body Mass Index, and Ponderal Index during the Study Period

During the study period of 14 weeks, no *S. suis*-associated disease occurred. Average daily weight gain from birth until weaning (236 g in the treated group versus 243 g in the control group) as well as in the nursery (456 g in the treated group versus 449 in the control group) did not significantly differ between treated and not treated pigs. No differences in body mass index and ponderal index at the different time points of measurement were observed between the two groups. Differences were determined by the student’s *t*-test for independent samples. Body mass index and ponderal index measured at weaning and at the end of the study were not influenced by the treatment (Table 2).

### 2.3. Prevalence of *S. suis* Colonization

**Figure 2:**
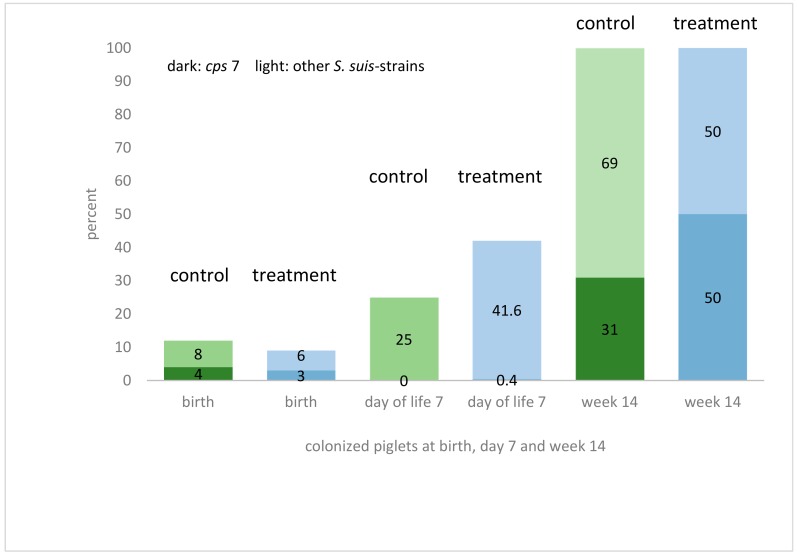
Detection rate of *S. suis* (dark green (control) or blue (treatment): *cps* 7, light green (control) or blue (treatment): other serotypes) at birth, day 7 of life and week 14 in the treatment and the control group in percent, detected on tonsils. Piglets in both groups were colonized almost equally when coming out of the birth channel before ceftiofur treatment. At week 14 all piglets were colonized on tonsils. No statistical differences could be seen between treatment and control group.

*S. suis* was cultivated from 84% of the pregnant sows from tonsillar swabs (16/19). Sixty-three percent of these isolates were determined to be *S. suis cps* 7 (*arc*A+*, gdh+, sly−, mrp−, epf−*), while the remaining isolates did not belong to *cps* 1, *cps* 2, *cps* 7, or *cps* 9 (also negative for the virulence-associated genes *sly*, *mrp* and *epf*). Two of the four gilts were found to be positive for *S. suis* on their tonsils. At birth, vaginal swab samples were positive for *S. suis* in 32% of the sows (6/19), but none was positive for *cps* 7. The same gilts which were negative in tonsils were also tested negative in the vagina. In the tested newborn piglets 21% were positive for *S. suis* either in their nose and/or in their tonsils. Positively-tested piglets were born by sows with or without positive findings for *S. suis* in the vagina during birth. On the other hand, some negatively tested piglets (*n* = 28 out of 37 tested piglets) were born by sows with a positive finding for *S. suis* in the vagina (*n* = 6). Positively tested for *cps* 7 (*mrp*−, *sly*−, *epf*−, *arc*A+) immediately after birth were seven piglets out of four different litters. Piglets of the future treatment group and control group were colonized almost equally (3% carriers of *S. suis cps* 7 and 6% carriers of other *S. suis* serotypes in the treatment group and 4% carriers of *S. suis cps* 7 and 8% carriers of other *S. suis* serotypes in the control group) (Figure 2). Noteworthy, *S. suis* strains isolated during the study period differed from those strains previously isolated from diseased animals at the same farm in the *mrp* genotype. Six days after treatment, *S. suis* was culturally detected in the tonsils of 42% of piglets in group A (treatment) and 25% in group B (control). Only one piglet (group A) was identified as a carrier of *S. suis cps* 7 (*arc*A+, *gdh+*, *mrp−*, *sly−*, *epf−*) (Figure 2). At the age of 14 weeks, all pigs were positive for *S. suis* in their tonsils. Fifty percent of the treatment group and 31% of the control group were positive for *S. suis cps* 7 (*arc*A+, *gdh+*, *mrp−*, *sly−*, *epf−*) (Figure 2). 

### 2.4. Bactericidal Humoral Immune Response and Cytologic Findings

**Figure 3:**
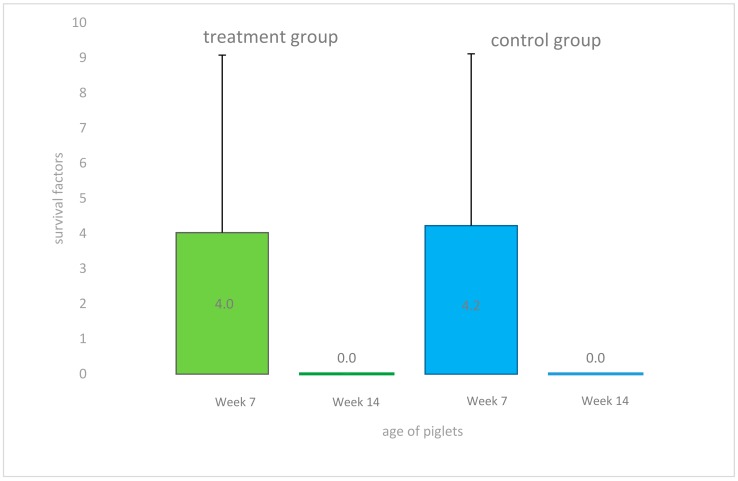
Results of the bactericidal killing assay using blood from *S. suis cps* 7 free piglets with sera collected at week 7 and 14 of life from piglets in the treatment and control group and the invasive *mrp*+ *cps* 7 strain. A survival factor < 1 indicates killing of *S. suis*, a survival factor > 1 indicates bacterial proliferation. No differences between treatment group and control group were seen in week 7 (*p* = 0.9) and week 14 (*p* = 0.1).

In weeks 7 and 14 of life, sera were collected from treated and untreated pigs and bactericidal activities of these sera against an invasive *S. suis cps* 7 strain isolated previously from a diseased pig of the study farm were determined (Figure 3). Noteworthy, bactericidal assays have become an important read out parameter in *S. suis* vaccination and pathogenesis studies as bactericidal activity in blood correlates with protection and virulence, respectively [34,35]. Changes in the bactericidal activity against a specific *S. suis* serotype in blood of a piglet is thought to be mainly due to the induction of opsonizing antibodies. In week 7 of life, bactericidal activity in sera (survival factor (SF) < 1) was only found in 15 pigs (41.6%) with a high variation between individuals (range SF 0–16.129) independent of the group. In general, piglets of the same litter had similar bactericidal activities three weeks after weaning, and were either all positive (SF < 1) or all negative (SF > 1). Two of the four gilt litters were positive (SF < 1) at that time, while the other two gilt litters were negative (SF > 1). However, in week 14 sera of all tested individuals (*n* = 38) demonstrated very high bactericidal activities (range SF 0.000–0.003). No differences in bactericidal activities were found between sera from treated and control pigs (week 7 *p* = 0.9; week 14 *p* = 0.1). Animals with and without bactericidal activities in sera were mixed in the nursery groups.

No differences were found between both groups with respect to red and white blood cell parameters during the course of the study with exception of the absolute (*p* = 0.02) and relative (*p* = 0.04) concentrations of eosinophilic granulocytes in week 14 of life which were decreased in treated pigs (Table 3).

### 2.5. Antimicrobial Susceptibility Testing

No resistance to ceftiofur (MIC ≤ 2 µg mL^−1^) [36] or another tested cephalosporine was seen in any isolate. All tested isolates (*n* = 36) were resistant to tetracyclin (MIC ≥ 2 µg mL^−1^) [36] and 19/36 isolates were resistant to trimethoprim/sulfamethoxazole (MIC ≥ 4/76 µg mL^−1^) [36]. 37.5% of the isolates from time of birth and 33.3% of the isolates from day 10 of life were resistant to trimethoprim/sulfamethoxazole. In week 14, only three out of eight isolates (all *cps* 7, *arc*A+, *gdh*+) were sensitive to trimethoprim/sulfamethoxazole, the other five isolates were resistant (75.0%).

## 3. Discussion

In accordance to previous findings of other authors [11,37], different *S. suis* strains were not only found at different time points, but also at the same point of time and in one individual animal. At least 20% of piglets were positive for *S. suis* in nose or tonsils immediately after birth, irrespective of previous positive findings in vaginal samples of the respective mother, which are in line with the results of Amass et al. [11]. Detection of *S. suis* in neonatal piglets just born from sows tested negative for the vaginal tract suggests that the results for the sows are false negative. Presumably, further sampling of sows might have been necessary to reduce the number of false negative sows. The impact of vertical transmission for herd health is still not clear, because it is not known, if the same pathotypes involved in the majority of herd problems after weaning had also been the early colonizers of the vaginal tract. Here, we confirmed early uptake of *S. suis* in newborn piglets and found that *cps* 7 strains are among the strains isolated from newborn piglets. However, these *cps* 7 strains were all *mrp* negative and thus different from the genotypes isolated previously from diseased piglets of this herd. Therefore, we could not show, that the invasive pathotype (*sly−*, *mrp+*, *epf− cps 7*) in this herd is also a frequent colonizer of the vagina. However, due to the limited sensitivity of the cultural approach missing few *sly−*, *mrp+*, *epf− cps 7* bacteria among all the other streptococci might be possible. Although in our study only minimal mixing was performed by merging only two to three litters and carrier rate before weaning especially of *S. suis cps 7* was low, transmission of *S. suis* peaked some time afterwards. The percentage of colonized animals at weaning is assumed to be a critical factor for outbreaks of streptococcal disease by some authors [38,39]. In addition, several management factors and the overall herd health status are considered to play a major role in disease outbreaks [40], though some authors were unable to correlate frequencies of carrier pigs, herd size, or management factors with disease [16,41]. This study was accompanied by changes in herd health management as omission of cross-fostering and specific stressors as tail docking and castration without anesthesia, less mixing, and a lower stocking density. All these measures might have had preventive effects and could have led to the reduction of clinical signs. The finding that at least three *S. suis* genotypes caused disease on this farm prior to this study might have been related to numerous predisposing factors allowing strains of moderate or even low virulence to cause disease. Despite the ceftiofur treatment at the age of 14 weeks, all sampled animals (*n* = 38) from all 19 litters (treatment and control) were positive, and from half of the piglets *cps* 7 strains were cultivated. Therefore, there is no difference to other studies, in which tonsillar isolation rates in piglets of this age group were as high as 98% [3] or in slaughter pigs as high as 100% [42]. At least for *S. suis cps* 2 it was shown, that it could colonize tonsils of carrier pigs for more than one year, even in the presence of circulating opsonizing and neutralizing antibodies [15,43]. During the course of this study, the virulent *S. suis* strain isolated from diseased animals on the same farm was not detectable. Generally, *S. suis* infections are endemic and herds might be affected with pathologies associated with a single *S. suis* pathotype for years (unpublished results of CB) [44]. However, there are other reports of disappearance of severe clinical *S. suis*-related recurrent diseases and their responsible strains in herds after a period of time for unknown reasons [13,25,45].

Interestingly, all animals developed bactericidal activity against the invasive *mrp*+ *cps* 7 strain until week 14, though this genotype was not detected in a single piglet in this study. This bactericidal activity is most likely due to opsonizing antibodies inducing phagocytosis and efficient killing by neutrophilic granulocytes [46]. As only one *S. suis* genotype was included in the respective assay, it is not clear how specific the registered bactericidal activity was. At this point it can only be speculated if subclinical infection of the piglets with a different genotype such as the frequently isolated *mrp*− *cps* 7 strain elicited these opsonizing antibodies or even a non-*cps* 7 strain eliciting cross-reactive antibodies. However, we can also not exclude that our cultural approach failed to detect the invasive *mrp*+ *cps*7 strain inducing this humoral immune response. It is, however, reasonable to hypothesize that all piglets investigated at 14 weeks of age were protected against the invasive *mrp*+ *cps* 7 strain, because this strain was efficiently killed in the blood of SPF piglets ex vivo only after addition of sera of these 14 week old piglets. Our results suggest that all piglets of a herd might go through an adaptive protective immune response against a prevalent *S. suis* serotype until the 14th week of life, even when the herd is not affected by *S. suis*-associated morbidity and mortality. 

Since there were no statistical differences between treated and control pigs with respect to frequencies of isolation of *S. suis* from the upper respiratory tract, early antimicrobial treatment does not seem to have an effect colonization with *S. suis*. This is in contrast to the results of Gascho et al. [33], who were treating not piglets, but pre-farrowing sows with ceftiofur and could find a significant decrease in number of positive sows at farrowing and a significant reduction in the number of positive piglets farrowed, nursery mortality, and number of individual treatments. Therefore, the main challenge for farmers and veterinarians is to decide about a point of time after recovery from a clinical outbreak when metaphylactic antibiotic treatment is stopped in sows and piglets. 

Ceftiofur long-acting preparations are used for metaphylactic treatment of piglets on many farms [29,47,48] and obvious side-affects seem to be rare. The decrease in eosinophilic granulocytes in treated pigs observed in this study might be a side effect, which will not become obvious under field conditions. In approximately 8% of human patients a mild and transient cephalosporin-induced eosinophilia is described [49]. 

Monitoring the antimicrobial use in Belgium revealed, that long acting ceftiofur was the second on the list of the most frequently applied injectable antimicrobials (40.1%). The 3rd and 4th generation cephalosporins ceftiofur and cefquinome were used in 48% of the visited herds [29]. In 2011, van Rennings et al. [47] analyzed the use of antimicrobials on 495 German farms and found beta-lactams among the most commonly used antimicrobials in piglets. Although the absolute amount of cephalosporins administered to swine is low in comparison to other substances mainly used in heavier pigs, they account for a relatively large proportion of treatment units. In Austria, 1% of the total amount of antimicrobials used in swine in 75 conventional pig farms was ceftiofur, which was mainly used for metaphylactic and prophylactic treatment in suckling and weaned piglets [48]. The advantages of modern antimicrobials like cephalosporins are the long and potent acting of low doses after a single injection [29]. It is highly recommended to culture respective *S. suis* isolates from affected pigs and perform resistancy testing before using antimicrobials, especially before using cephalosporins [50,51]. A high proportion of *S. suis* isolates is resistant to tetracyclines worldwide [52]. This is reported in detail from several countries, such as in Denmark with 52% resistant strains and 30% resistant *S. suis cps* 7 isolates [27], Spain (95%) [53] or Italy (90%) [54]. In the present study 75% of the isolates were resistant to tetracyclines. The prevalence of *S. suis* isolates resistant to penicillin varies between countries, but is generally low. In Canada, 0–27% of *S. suis* isolates were resistant to penicillin, ampicillin (0.6–23%), and ceftiofur (0–23%) [52]. All Danish *S. suis cps* 7 strains isolated by Tian et al. [27] were susceptible to ceftiofur. Similar to the present results are those of Van Hout et al. [55]: 0.5% of 1163 Dutch isolates from clinical submissions were resistant to ceftiofur, 3% were resistant to trimethoprim/sulfamethoxazole, and 78.4% resistant to tetracyclines.

In this study all *S. suis* isolates were susceptible to ceftiofur, but treatment had no detectable beneficial effect on piglets’ health. Average daily weight gains, as well as carrier status for *S. suis* were similar in treated and untreated pigs. 

As colonization and transmission of *S. suis* was not influenced by ceftiofur treatment in this study and bactericidal humoral immunity developed similarly in both groups, the hypothesis, that early treatment with ceftiofur will hamper the rise of opsonophagocytic antibodies, is rejected. Treatment of clinically healthy piglets should be accompanied by general measures minimizing transmission of the pathogen and avoiding stressors and should not be continued as a routine measure on the farm, because the potential beneficial effects on the health of the piglets are overestimated, as indicated by the results of this study.

## 4. Material and Method

### 4.1. Study Farm, Animals, and the Design of the Study

The experimental protocol was approved by the Animal Ethics Committee of the University of Veterinary Medicine Vienna and the Austrian Federal Ministry of Science and Research according to the Austrian Animal Protection law (BMWF: 41.3-63003—01/2013). 

**Figure 4:**
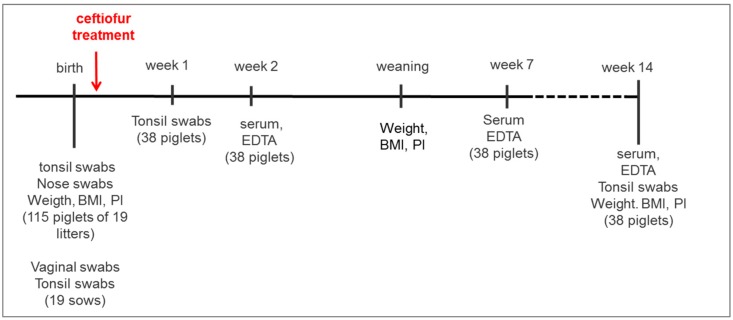
Time flow of the study. Piglets were weaned at four weeks of age.

Fifteen sows and four gilts, which were in a good health status and had not been treated with antimicrobials during pregnancy, were included in the study and were not treated with antimicrobials throughout the whole lactation period. Five days prior to the expected farrowing date, sows were washed with soap and moved to well-cleaned and disinfected individual free farrowing pens. Tonsillar swab samples for bacteriological diagnostics were collected from all sows right before farrowing. Birth was supervised continuously, so that piglets could be removed manually from the vagina using sterile obstetrical sleeves avoiding contact with the environment. Immediately, sterile gauze was used to remove fetal membranes covering each piglet’s rostral surface before tonsillar and nasal swabs were obtained from every single piglet and piglets were ear-tagged immediately afterwards. In total, 115 piglets were included in the study (Figure 4). Two hours after the first piglet was born in each litter, a deep vaginal swab was taken from the respective mother sow (*n* = 19). In case of vaginal discharge during the lactation period swab sampling was repeated in the respective sow. Approximately 12 h after the beginning of farrowing, male and female piglets within each litter were separated, weighed and the crown-rump-length was measured for calculation of the body mass index (birth weight/(crown-rump-length)^2^) and ponderal index (birth weight/(crown- rump-length)^3^) [56]. Using simple randomization, half of the females and males of each litter (group 1) received a single intramuscular injection of a vegetable oil-based suspension of a ceftiofur crystalline free acid in the recommended dose of 5.0 mg/kg BW (Naxcel^®^, Zoetis Belgium SA, Louvain-la-Neuve, Belgium), whereas the other half (group 2, control piglets) received an intramuscular injection of sterile 154 mM sodium chloride (0.2 mL/kg BW; NaCl 0.9% B.Braun^®^, B.BRAUN Melsungen AG, Melsungen, Germany). Health status of the animals was supervised daily and piglets were not cross-fostered but stayed with their mothers during the whole lactation period. Tonsillar swabs of one treated and one untreated randomly chosen piglet per litter (*n* = 38) were taken at day 7 and in week 14 of life. Whole blood and serum samples of one treated and one untreated randomly chosen piglet per litter (*n* = 38) were collected by puncture of the jugular vein (Primavette^®^S and Kabevette^®^ (KabeLabortechnik GmbH, Nürnbrecht-Elsenroth, Germany) in weeks 2, 7, and 14 of life, respectively. After coagulation, serum samples were centrifuged for 10 min at 1500× *g* and serum was transferred into 1.5 mL tubes and stored at −20 °C until further use. The EDTA stabilized blood samples were analyzed within two hours after sampling in an automatic cell counter (IDEXX Procyte DX, IDEXX, Stuttgart, Germany) for hematological indices (erythrocytes (RBC), hematocrit (HCT), hemoglobin (Hb), mean cell volume of erythrocytes (MCV), mean corpuscular hemoglobin (MCH), mean cell hemoglobin concentration (MCHC)), platelets, and white blood cells (total cell count, neutrophils, lymphocytes, monocytes, eosinophils, basophils).

Neither tail docking nor teeth-grinding was performed. Piglets were castrated in week two of life under general anaesthesia with azaperone (0.5 mg/kg BW i.v.) and ketamine (10 mg/kg BW i.v.). After weaning, two to three litters were combined in one pen in the nursery and no further mixing of animals was performed until the end of the study. Piglets were weighed at weaning (28 days of life), in week 11 before entering the fattening unit and in week 14 at the end of the study. Piglets included in the study were not treated with antibiotics until the end of the examination period (Figure 4). 

### 4.2. Bacteriological Examination

Swab samples taken were maintained at 5–7 °C until arrival at the Institute of Microbiology, University of Veterinary Medicine, Vienna, plated onto Columbia Agar with 5% sheep blood (BBL, BD Diagnostics, Schwechat, Austria) and incubated aerobically within 24 h after sampling. After 24 h of incubation at 37 °C, α-haemolytic colonies were Gram-stained and characterized using biochemical tests (API, bioMérieux, Wien, Austria). All *S. suis* isolates were sent to the Institute for Bacteriology and Mycology, Faculty of Veterinary Medicine, Leipzig, Germany und further characterized by multiplex PCR [24] for the housekeeping gene *gdh,* the genes *cps 1, cps 2, cps 7*, and *cps 9* encoding serotype specific enzymes involved in capsule biosynthesis (here referred to as *cps* 1, *cps* 2, *cps* 7, and *cps* 9) and for the genes *epf*, *mrp*, *sly* and *arc*A encoding for virulence-associated factors extracellular factor, muramidase-released protein, suilysin, and arginine deiminase. 

The antimicrobial susceptibility of *S. suis* strains was determined using broth microdilution according to approved standards of the Clinical and Laboratory Standards Institute (CLSI) [57]. Minimal inhibitory concentrations (MICs) of 12 antimicrobials were determined employing in-house 96-well MIC plates containing serial two-fold dilutions of penicillin (0.063–32 µg/mL), ampicillin (0.063–32 µg/mL), amoxicillin/clavulanic acid (0.125/0.063–32/16 µg/mL), cefepime (0.063–8 µg/mL), ceftiofur (0.063–8 µg/mL), cefquinome (0.063–8 µg/mL), enrofloxacin (0.063–8 µg/mL), marbofloxacin (0.063–8 µg/mL), tetracycline (0.125–16 µg/mL), erythromycin (0.125–8 µg/mL), clindamycin (0.25–4 µg/mL), and trimethoprim/sulfamethoxazole (0.063/1.188–8/152 µg/mL). Isolates were reported as susceptible, intermediate, and resistant using CLSI veterinary breakpoints if available [36]. For clindamycin and trimethoprim/sulfamethoxazole human-derived breakpoints were applied [36].

### 4.3. Bactericidal Killing Assay

Bactericidal killing assays of *S. suis* in the presence of 20% (*v*/*v*) porcine serum was assayed essentially as described by Unterweger et al. [22] and Baums et al. [45]. Briefly, heparin blood of pigs free of *S. suis cps* 7 was mixed with the serum sample to be tested and inoculated with the *S. suis cps* 7 strain isolated from a pig with meningitis and arthritis during the former disease outbreak on this farm. Serum samples from treated and untreated piglets in week 7 and 14 of life were tested. Before and after a 120 min incubation time at 37 °C serial dilutions were plated onto blood agar to determine colony forming units (cfu) after overnight incubation at 37 °C. The survival factor (SF) is defined as the ratio of the cfu after incubation to the cfu at the beginning of the two-hour incubation time. 

### 4.4. Statistical Analysis

Statistical analyses were performed using SAS^®^ software, Version 9.3 (SAS Institute Inc., Cary, NC, USA). Differences in frequencies of positive bacteriological findings for *S. suis* between treated and untreated animals were determined by the Fisher’s Exact Test. Quantitative parameters as red and white blood cell counts, average daily weight gain, and SF were tested for normal distribution by the Shapiro Wilks Test. Differences between treated and untreated animals in normally distributed data were determined by the student’s *t*-test for independent samples. In not normally distributed data the Wilcoxon test was used for group comparison. Correlations were identified by Spearmen’s rank correlation coefficients (not normally distributed data).

## Figures and Tables

**Table 1:** *S. suis* strains isolated in brain/cerebrospinal fluid and joints in acute diseased piglets, clustered in three different groups.

Group	Numbers of Isolated Strains	*S. suis* Serotype	*epf*	*mrp*	*arc*A	*sly*	Location of Isolation
1	3	*7*	−	+	+	−	meninges, cerebrospinal fluids
2	2	*? (not 1,2,7,9)*	−	+	+	+	meninges, cerebrospinal fluids
3	2	*? (not 1,2,7,9)*	−	+	+	−	meninges, joints

**Table 2:** Mean body mass index (BMI) and PI (ponderal index) in control and treatment group at weaning and in week 14 (N (control and treatment, respective) = 84; NS: not significant, *p* > 0.05).

Group	Weaning		Week 14		*p*
	BMI	PI	BMI	PI	
Control	30.1	62.9	49.1	84.2	NS
treatment	30.4	64.7	49.3	84.4	NS

**Table 3:** Mean red and white blood cell parameters (ery: erythrocytes, Hct: hematocrit, hb: Hemoglobin, leu: leucocytes, neu: neutrophile granulocytes, eos: eosinophilic granulocytes, bas: basophil granulocytes, lym: lymphocytes, mono: monocytes; not all data shown) in treatment (T) and control (C) group at week 7 (A) and week 14 (B). * *p* = 0.023; ** *p* = 0.041.

Group	Time of Measure	ery	Hct	hb	leu	neu	eos	eos	bas	mono	lym
		×10^12^/L	%	g/dL	×10^9^/L	×10^9^/L	×10^9^/L	%	×10^9^/L	×10^9^/L	×10^9^/L
T	A	6.132	34.279	10.229	13.495	4.986	0.163	1.217	0.008	0.580	7.757
	B	6.988	37.738	11.557	30.643	9.543	0.125 *	0.662 **	0.015	0.579	10.066
C	A	6.172	33.300	9.963	14.583	5.727	0.138	1.041	0.008	0.598	8.111
	B	6.962	38.400	11.711	24.075	9.263	0.147 *	0.447 **	0.043	0.875	13.279
